# The Incompatibility of Motherhood and Professional Women's Football in England

**DOI:** 10.3389/fspor.2021.730151

**Published:** 2021-09-30

**Authors:** Alex Culvin, Ali Bowes

**Affiliations:** ^1^Salford Business School, University of Salford, Salford, United Kingdom; ^2^Department of Sports Science, Nottingham Trent University, Nottingham, United Kingdom

**Keywords:** football, gender, motherhood, policy, professional, work, football (soccer), gender

## Abstract

There has been an advancement of professionalisation of women's football globally. Professional women's football in England has grown exponentially since the inception of the Football Association (FA) Women's Super League in 2011. This article offers an examination of the gender-specific needs of women as professional footballers, focusing specifically on the distinctive aspect of maternity policy. Sportswomen often feel compelled to make the difficult decision between motherhood and an athletic career. Contracts have become an essential component of the work-life of professional women footballers. However, these contracts pose questions regarding organisational intentions and motivations. This study draws on 30 semi-structured interviews with professional women footballers in England. Our key considerations include contracts, employment, motherhood, and athletes' bodies. This examination will develop our understanding of the complexities associated with professional football as a career choice for women.

## Introduction

This article examines the distinct employment conditions of professional women footballers in England. Specifically, we explore the gender-specific contractual requirements of professional women footballers in relation to maternity policy through the examination of player interpretations and the understanding of motherhood and their bodies in relation to contractual employment in professional football. Women's football has evolved rapidly during the last decade, with the 21st century bringing an increasing number of professional leagues for women (Culvin and Bowes, 2021). However, the sustainability of professional leagues for women has been interrogated due to league failures (Allison, 2016). Despite questions of durability, in 2011, the national governing body for football in England, the Football Association (FA), launched the FA Women's Super League (FA WSL). Initially, a semi-professional league, the league got fully professionalised in 2018, making football a legitimate career opportunity for elite women footballers in England. However, the voices of women articulating their experiences as professionals are notably absent from the current research literature (Taylor et al., 2020).

Despite the gender-specific workplace requirements for women related to maternity, to date, no empirical studies have explored either maternity policies and contracts or the experiences of players regarding maternity in professional women's football. In addressing this, we explored the workplace experiences of FA WSL footballers in relation to their professional contracts, specifically considering their understanding of maternity policy, and motherhood. Although football organisations are increasingly taking the development of the women's game more seriously (Andersson and Barker-Ruchti, 2018), it is only recently that stakeholders in the game developed regulations designed to protect female professionals during maternity, coming into effect on January 1, 2021 (FIFPro World Players' Union, 2020). The aim of this research, then, was to gain an understanding of the distinctiveness of career development and the contractual conditions and requirements of women entering this new profession. Moreover, to identify the workplace conditions and requirements of professional women footballers in England, and to provide evidence to shape gender-specific policy and contribute to more gender-equitable approaches, especially in relation to maternity, in elite sport. Having outlined the intricacies of football as work, the sections of analysis in this study highlight the unreported and overlooked aspects of contracts, employment, and motherhood in relation to the careers of professional women footballers in England.

To contextualise the study, we need to examine a number of associated themes in relation to football as work. We begin by first setting out the development of professional women's football in England, considering football as a professional occupation before turning to maternity as a gender-specific consideration for women professional athletes. A discussion on the intricacies of professional contracts and policy in sports, with respect to maternity, follows before we consider the consequences of neglected policy in the case of women professional athletes.

### Football as Work

Although there have been various iterations of the professionalisation for women's football in multiple contexts, most notably in the United States (Allison, 2018), the inception of the FA WSL in 2011 created the first widespread opportunity for football as work in England for elite women footballers. The inaugural FA WSL season comprised eight teams and a short summer season, with the introduction of a semi-professional summer league aiming to provide a commercially viable, competitive product. However, in their discussion on the structure, governance, and impact of the FA WSL, Dunn and Welford (2015, p. 92) argue that the placing of women's football as a summer league effectively rendered them “outsiders on the inside.” The development of elite football for women was central to FA objectives and marked a shift from the much-discussed FA ban on women's football that spanned between 1921 and 1971. However, in light of the interest of FA in developing the game, scholars were critical of the organisation's marketisation methods concerning women's football, which appeared to be based primarily on commercial business objectives (Bell, 2012; Dunn and Welford, 2015). Furthermore, decisions around the development and structure of the FA WSL were significantly opaque, but the goal of the FA was for clubs to become small, sustainable, and professional businesses (Woodhouse et al., 2019). Consequently, two critical considerations emerged, which would influence contractual policy for players and, by extension, the experiences of players with professionalisation. First, increased investment resulted in the heightened professional expectations of both clubs and players. Second, professionalisation processes were accelerated, meaning a disparity emerged between clubs who could financially and structurally invest more and those who could not. In 2018/2019, the league had transitioned to a traditional winter season and had fully professionalised into a 12-team competition. New criteria for teams in the professional FA WSL era were included a minimum of 16 h of contact per week for players, a minimum investment per club, an academy as part of the club, and financial fair play regulations including a salary cap (Garry, 2017). This has led to some teams thriving and others struggling to meet the specific requirements. More recently, a landmark £7 million broadcasting deal was announced for the FA WSL (Clarkson et al., 2021). As such, the development of the FA WSL can be considered complex, and identifies football as an emerging, yet uncommon, and precarious occupation for women in England. Although more widespread opportunities for women in professional football are emerging in a variety of contexts, women's football still suffers from a lack of opportunities and resources in order to sustain and preserve this new profession.

Fédération Internationale des Associations de Footballeurs Professionnels FIFPro World Players' Union (2020, p. 6), the world representative for 65,000 professional footballers, explains that “viable career paths and proper working conditions are still lacking” in the women's game, and although the sport is increasingly professionalising, “adverse labour conditions still plague the game.” This can be evidenced in research related to pay, contracts, and working conditions. Drawing on the 2017 FIFPro Global Employment Report, some of the key concerns related to the employment of women in football centres on pay and contracts specifically. The report highlighted that 50% of elite women footballers receive no pay, and almost two-thirds of those who do receive a salary earn < $600 per month, with that figure representing the average salary of the global women footballer. Only “a tiny few” made more than $4,000 a month (p. 14). The average contract length in 2017 was 12 months, although only 47% of players had an employment contract. Professional players felt they were not always recognised as such by stakeholders in the game, and, interestingly, by themselves. Fédération Internationale des Associations de Footballeurs Professionnels FIFPro World Players' Union (2017) identify that this perception can mean that the players are not given the appropriate rights and protections and are often unaware of their rights. In the survey, 43.8% are identified as amateurs, 32.1% are identified as semi-professionals, and only 24.1% are identified as professionals. Furthermore, FIFPro World Players' Union (2017) also highlighted that only 18% of women were professional according to the Fédération Internationale de Football Association (FIFA) Regulations, the international governing body for the sport. This includes having a written contract and being paid more for their footballing activity than the expenses incurred. By 2020, it was reported that monthly club salaries had increased for women players, but that 3.6% of players at the Women's World Cup, the pinnacle of the sport, were not receiving any money to play (FIFPro World Players' Union, 2020).

Whilst the increased professionalisation of the women's game is decidedly recent, Roderick (2006, p. 1) indicates that the men's professional game developed in the nineteenth century, and the job of a (male) professional footballer has emerged into a “relatively prestigious occupation.” It is fair to say that we cannot make those assumptions about women in the same profession. Despite the relatively prestigious nature of men's football as an occupation, the contribution made by Roderick (2006) to the understanding of professional football as work also highlighted the fragile and uncertain nature of playing careers. Football is a labour-intensive industry, with an increasingly mobile labour force (Roderick and Schumacker, 2017). The fragile labour market realities for players with limited transferable employment capital mitigates against stable working conditions (Roderick and Schumacker, 2017). The unique skills and talent of players are not easily reproduced, and their salaries tend to reflect the “capital” of individual players (Wacquant, 1995). However, Roderick (2006) argues that professional football at all levels can be materially rewarding. This view can be considered male-centric, as only a small proportion of elite women footballers have the opportunity to sustain themselves adequately (FIFPro World Players' Union, 2017). Indeed, the capital or value of a player can change dramatically if they sustain a long-term injury or, for women professionals, become a mother (Szymanski, 2010). Individualised *risk biographies* (Beck, 2000) mean uncertainties, such as injury or pregnancy costs, which are borne by the individual rather than the employer or the state (Sennett, 2006).

Despite figures suggesting that less than half of professional women footballers have employment contracts, the sporting contract of an athlete is considered a necessary part of their career; as such, they have received increased scholarly attention in recent years (Kohe and Purdy, 2016). Wong et al. (2010) suggest that contracts represent more than financial incentives for athletes, such as prestige, legitimacy, and popularity, which can all motivate athletes. For the most part, contracts formalise the relationship between the athlete and the club. To secure a contract, athletes must not only perform in training and competitively, but also conform to organisational demands (Kohe and Purdy, 2016); although, arguably, the structural conditions that influence employment contracts in football are inherently gendered. Clearly, as women's sport professionalises, there are problems that are increasingly evident. The current lived realities for many elite women footballers are an existence that has only short-term security, financial precariousness, and inadequate resources. These circumstances have a significant impact on the athletic careers of women in terms of both early retirement and decisions about motherhood (FIFPro World Players' Union, 2017).

### Professional Women Athletes, Motherhood, and Maternity

The number of professional women footballers who are mothers in the professional game is particularly low and is indicative of mothers in elite sports more broadly. A survey completed by over 540 elite female athletes in Norway indicated that the number of mothers was only 4% (Bø and Backe-Hansen, 2007). Globally, data from FIFPro World Players' Union (2017) state that from 3,295 women footballers surveyed, only 2% of respondents were mothers and 47% felt they would have to leave the game early to start a family. The report highlights that 61% of women players are offered no childcare support, only 8% of players who had children by 2017 were provided maternity pay from their National Governing Body or their club, and only 3% of clubs provided childcare support (FIFPro World Players' Union, 2017). As FIFPro World Players' Union (2017, p. 4) states in an attempt to address this problem, “appropriate benefits and protections for players to become mothers must be standardised across the women's professional game, and the proper training conditions must be in place to offer players the opportunity to return to their peak performance after giving birth.”

In response to some of these challenges, in 2020, FIFPro published their Player Pregnancy and Parental Management report, in line with the International Labour Organisation (ILO) convention number 183 from 2000. The policy guide outlined directions in both mandatory and guided forms for players and organisations on the contractual requirements and obligations for pregnancy and maternity, aiming to protect players against discrimination. Key mandatory points include rights when pregnant, parental leave, and return to play. Key guided points include parental management and travel and non-discrimination: pregnancy and parental policy. Whilst this policy guide is important and noteworthy, it remains to be seen whether Member Associations and clubs specifically implement this policy and, if not, what the repercussions are for the unimplemented policy.

Specifically, in relation to the FA WSL, there are increasing discussions around pregnancy and motherhood. Emma Hayes (the current manager of Chelsea Women FC) described the lack of childcare support offered to players as a disgrace (Dean, 2017). In 2020, Manchester United and England goalkeeper Siobhan Chamberlain spoke out about her experiences as a mother playing professionally in the FA WSL, stating that her contract did not include maternity benefits and she had to request additional leave and pay from her club (McElwee, 2020). Comparatively, Taylor et al. (2020) identify six sports in Australia that offer maternity and childcare support to their athletes, including, the Australian Women's Football League, basketball, cricket, football (W-league), netball, and rugby union. However, when beginning to understand the difficulties professional women footballers experience at work in England in relation to their maternity rights, these complications can be attributed in part to a lack of female representation in decision-making positions in the sport. The gendered space of sport workplace settings has been recognised in the literature (Burton, 2015), alongside an acknowledgment that women are largely underrepresented in the sport workplace (Acker, 2010). According to Adriaanse (2016), a critical mass of 30% of women on boards must be achieved in order to influence the organisation. Importantly, from 45 countries analysed, only four achieved critical mass. Specific to football, Bradbury et al. (2011) note that there are low levels of women in the regional, national, and European levels of governance in football and a lack of organisational provision for women and girls. Taken together, the core difficulty, then, is the underrepresentation of women in decision-making positions, which means that the experiences of women are overlooked. Lacking gender-specific policies such as maternity, organisations and teams respond with poorly conceived ideas around women and pregnancy. Thus, we can theorise that male-dominated football organisations and clubs ignore the biological differences between men and women with respect to pregnancy, despite the physical process of pregnancy and childbirth having a much bigger impact on female athletes.

Becoming a parent is one of the most significant experiences in life, but this transition phase is notably under researched in athlete-as-worker research (Tekavc et al., 2020). However, there is a small but growing body of research dedicated to the exploration of elite women athletes and motherhood (Appleby and Fisher, 2009; Douglas and Carless, 2009; McGannon et al., 2018; Darroch et al., 2019; Tekavc et al., 2020). The discursive discourse surrounding pregnancy and maternity is fairly contradictory (Darroch and Hillsburg, 2017). Women contend with reinforced common-sense constructions that stress social roles, with women as primary caregivers, which often mean women putting their careers on hold whilst they raise a family (Morgenroth and Heilman, 2017). However, the careers of athletes are short, as such, there is a perceived incompatibility with women having a family during their athletic career (Roberts, 2018). Many women feel that starting a family must come at the end of their athletic careers (Tekavc et al., 2020), unlike men who can often balance parenthood with sports (Tekavc et al., 2015).

Previous research on the experience of women with maternity, pregnancy, and return to sports post-partum overwhelmingly focuses on individual sports, including long-distance running and golf. Pedersen's (2001) research on the identities of Danish international athletic mothers demonstrated a perceived clash between home life and elite sports, whilst research on elite athlete mothers in New Zealand competing in both team and individual sports highlights the necessity of organisational policies, such as child-care resources and networks, to support their return to sport (Johnston and Swanson, 2007). More recently, Tekavc et al. (2020) highlight the careful planning involved in pregnancy, with athletic careers of women dictating the time women conceive. This body of research suggests a meshing of cultural discourse and sports constructs elite mother athlete identities and their associated meanings in different ways (Douglas and Carless, 2009; McGannon et al., 2018).

Research that explores the experiences of elite athlete mothers highlights how mothers navigate gendered ideologies, such as the expected behaviours of a “good mother,” which can make mothers vulnerable to guilt and psychological distress if they cannot live up to expectations (Appleby and Fisher, 2009; McGannon et al., 2018). In this regard, research has found that elite athlete mothers experience extreme guilt when returning to their sport, as competition schedules dictate that athletes spend much time away from home, thus missing the milestones of their child and so on (Palmer and Leberman, 2009). Furthermore, if mothers do not reproduce expected social norms, they risk being labelled as selfish or bad mothers (McGannon et al., 2012). For women in individual sports, motherhood meant women devoted less time, although with increased efficiency, to training (Tekavc et al., 2020). Tekavc et al. (2020) also reported a concern that women may lose financial support during pregnancy, with Darroch et al. (2019) highlighting practises by both governing bodies and corporate sponsors that discriminate against pregnant women athletes. It is perhaps unsurprising then that women athletes often perceive there to be an incompatibility between athletic careers and pursuing motherhood.

### Motherhood, Contracts, and Policy

Given that an increasing number of women in team sports are operating in professional environments and are formally contracted (Bowes and Culvin, 2021), issues around pregnancy and maternity become both paramount and complex. For some, a maternity policy does not feature as a contract obligation, and for others, maternity leave has historically been underpaid, for example, the developments in the Women's National Basketball Association collective bargaining agreement (Mertens, 2020) and the maternity battle of Allyson Felix with Nike (Barr, 2019). Maternity is, however, increasingly being placed on the agenda for sports organisations. Yet, even if organisations do include maternity as a contractual consideration, some women in sports do not feel able to utilise this policy. Dixon et al. (2008) reported on intercollegiate athletic departments in the US, wherein many women felt that, if they used gender equity policies, such as taking full maternity leave, they would be perceived negatively by co-workers, potentially hindering their progression within the organisation.

Whilst it is important for organisations to have policies in place to reconcile the work-life experiences of women and other marginalised groups, efforts must be made to remove the negative stigma attached to using policy. Morgenroth and Heilman (2017, p. 56) note that:

Women who chose to take maternity leave were viewed as having work/family priorities that conformed to descriptive gender stereotypes and suffered in their work-related evaluations, and women who chose not to take maternity leave were viewed as having work/family priorities that violate prescriptive gender stereotypes and suffered in their family-related evaluations.

However, previous research suggests that the creation and implementation of family-friendly policies can have a positive effect on organisations, including men (Burton, 2015). Universal support for families, such as childcare and paid family leave, are common throughout Western Europe (Hegewisch and Gornick, 2011). Contradictions and tensions exist as gendered structures remain incredibly resilient, despite changing gender roles, relationships, and the increased implementation of workplace policies. Thus, workplace policies designed to encourage flexible work practises can often reproduce inequalities, as it is women who are more likely to utilise policy that can perpetuate inequalities both in work and non-work settings (Morgenroth and Heilman, 2017). Unsurprisingly, then, the notion that athletehood and motherhood are incompatible persist within the sport, exemplified by instances, such as challenges to training and recovery (Tekavc et al., 2020), a lack of institutional support, and reductions in sponsorship (Darroch et al., 2019).

### Professional Football and Women's Bodies

Evidently, a job as a professional athlete is one that is heavily reliant on the body of an athlete. A career in football is considered to be highly focused on body monitoring (Roberts, 2018) and intense in its approach to quantification, accountability, and surveillance (Roderick et al., 2017). Moreover, many women athletes are placed under pressure to subscribe to both an athletic ideal within their sport and a feminine ideal in broader society. Papathomas et al. (2018) label this as a paradox for women athletes. Such an environment may cultivate a loss of autonomy for athletes, as physical markers, such as athleticism or looking athletic, distinguish excellence, adherence, and commitment to professional status, which is perhaps absent in other forms of employment and/or retirement (Manley et al., 2016).

Football managers are able to exercise control over their players through coveted capital, for example, in the form of contract extensions, the building of the reputation of players, and game day selections (Roderick, 2006). Consolidating a position within the starting 11 can be considered a high priority for all professional footballers. In this way, uncertainty is a central feature in the lives of professional footballers, with career advancement never secure; thus, these players are operating in a hazardous career, whereby are only as good as their last game (Roderick, 2006) and physical capital is central to their work. Furthermore, the employment concerns of many women players potentially emerge from proving their worth as professionals. This includes, in England, *inspiring a generation*, a tag line created by the FA to increase participation figures (The Football Association, 2018). A failure to *produce the goods* can leave players vulnerable to feelings of inadequacy and incompetency. Therefore, it can be assumed that players would be willing to sacrifice almost anything to ensure their position in the football field, for example, playing whilst injured, 21 delaying family, or accepting a less than satisfactory work environment. Should injury prevent athletic involvement, it can invoke a loss of identity (Lockhart, 2010), with Darroch et al. (2019) highlighting female athlete's pregnant bodies are often framed in the same way as obtaining injuries, considered deficient.

Footballers depend heavily on their embodied assets and are prone to occupational destitution through the erosion of their bodily capital (McGillivray et al., 2005). To preserve bodily capital, footballers are encouraged to follow intense practises of eating, sleeping, and resting to ensure bodily care. In this sense, motherhood, and family life, with its impact including a reduction in time spent resting and training (Tekavc et al., 2020), is in conflict with the expectations of football clubs and significant others (coaches, managers, and agents) who largely influence the careers of players. According to Roberts and Kenttä (2018), this perceived incompatibility is often a reality, as athletes navigate inherently patriarchal workplaces that are both intolerant and underprepared for athletes as mothers.

Outside of sports, efforts have been made to explain the reluctance of employees to voice their opinions on important issues such as workplace marginalisation through policy (Manley et al., 2016). Research suggests employees are unlikely to voice their opinions if the environment discourages such communication (Morrison and Milliken, 2000) and/or is likely to be damaging to career progression (Donaghey et al., 2011); both apprehensions can be considered to exist within professional football clubs (Roderick, 2006). Women have the additional bind of the expectation that they are grateful for the opportunity to operate in professional sports settings, no matter how unsatisfactory they are (Culvin, 2019; Pavalidis, 2020; Taylor et al., 2020). Whilst the body of research primarily draws upon the experiences of individual athletes, this study aimed to draw upon the experiences of professional women footballers as they described the challenges associated with motherhood and a career in professional football.

## Methodology

This specific study draws upon data collected as part of a larger doctoral project, which analyses football as work for women in England. The *thinking tools* of Bourdieu were employed in the doctoral project, which enabled a relational understanding of the complex and precarious workplace and work-life conditions of professional women footballers (Jenkins, 1992). Bourdieu sought to bridge the dichotomy between structure and agency by integrating an analysis of the experiences of social agents and the scrutiny of the objective structures that permit those experiences in the first place (Jenkins, 1992). Utilising a relational framework allows the objects under investigation to be seen in context as part of the whole. Bourdieu contributed to sociological ventures and was particularly influential in exploring dynamic social practises, dominant power relations, and influential factors which contribute to (re)producing unequal relations in society. This study employs a qualitative interpretative framework that allows for an in-depth understanding of football as work for women in England. This qualitative research draws on semi-structured interviews with 30 professional women footballers completed by the lead researcher. Like previous research that has attempted to explore and describe hard to reach social fields (Roderick, 2006), and avoiding the subject or object dualism, which is key to the work of Bourdieu, this section includes first-person accounts of the lead researcher.

Footballers, on both a national and international stage, are not always willing to give access to their lives and are a notoriously hard group to reach (Roderick, 2006). As a former professional footballer, myself, it was important to present my position in the social field and relate experiences I had during my football career to the research process. My experiences at different clubs and my biography as a player shaped my research ambitions, particularly the emphasis on employment policy and the welfare of players. There were times I understood player welfare to be last on the list of concerns for clubs. Scholars support this notion, suggesting our interests have guided our decisions before the research is conducted (Sparkes and Smith, 2014). My life and experiences as a footballer underpinned my ambitions for the research, and more than this impacted my thoughts and feelings towards the object of my research, the players. I set out as a footballer insider, turned researcher outsider.

Considering the position of the researcher then, methodologically, this research project adopted an interpretative qualitative approach, which is the approach most appropriate given the “insider position” of the researcher (Merton, 1972). The interpretative paradigm is informed by the concern to understand the social world as it is (Morgan, 1980). For the interpretative researcher, the world of our lived experience, the lifeworld, and life history are the fertile ground upon which our understandings grow and formulate (Angen, 2000). Our very being of individuals in the world means that we are already morally implicated.

### Participants

Following institutional ethical approval, a criterion-based sampling strategy for participant recruitment was adopted, whereby participants were chosen because of a particular characteristic or feature (Patton, 1990). The criterion was that all participants must be women playing currently in the FA WSL at the time of the interview, between 2017 and 2018. Given the personal network of the lead researcher, requests were made directly to players *via* social media, email, or SMS, and all those who responded were interviewed. The ages of participants ranged from 20 to 34. Twenty-seven participants were full-time professionals, two were considering a full-time contract, and one was in a dual career. At the time of the interview, the data set comprised 15% of the total players in the FA WSL and, of that, 6.67% were mothers. The average age of the players who were interviewed was 25.4 years and, from the 30 players, 25 had played international football at either the senior or youth level. Senior internationals are defined as players who have represented the senior international team and youth internationals are players who have represented youth teams under 23's to under 19's. As part of a bigger PhD project, the interview schedule was constructed and divided into six sections: demographic information, football-specific information, dual career or education, life in the Women's Super League, contracts and policy, and life as a player or identity and future. The questions attempted to guide players through accounts of their life history from youth football to securing professional status, their experiences of life as a professional and how they understand football as work. Relevant to this study are the discussions on the experiences of playing football as professionals, and how football was understood as work.

Interviews were recorded after informed consent had been given by the participants. Previous research suggests football players have fears of openly criticising their managers or teammates (Roderick, 2006). Similarly, professional women athletes have noted that they have a fear of punishment for speaking critically and feel an expectation to speak positively and be grateful for their involvement in elite, professionalised sports (Bowes et al., 2020; Pavalidis, 2020). With this in mind, it was important to reassure players of anonymity and confidentiality, as their responses to questions would almost certainly involve not only their coach and teammates but also their clubs and the FA. It was important for players to know their comments would not be traceable to them to enable a free space for responses. The interviews lasted approximately between 45 and 150 min.

### Data Analysis

The data analysis process was a long-term project, with the lead researcher initially utilising an immersive, thematic approach:

Throughout the course of the research process, I identified emerging patterns within the data. I followed several steps to analyse the data affording the necessary primacy to participants' own understandings of their experiences as professional footballers. To gain a sense of familiarity with the transcript, I read, and re-read each transcript. I made initial comments on each transcript, fairly loosely in terms of what I deemed to be significant. The comments I made involved one-word comments, descriptive content, and the conceptual ideas of Bourdieu. Certain themes emerged, and each theme was identified and labelled. At this point, I was essentially attempting to capture what was said and make theoretical and practical connexions across participants. The themes identified in the first instance helped orient the second and so forth. This process resulted in 21 substantive themes being identified. The subthemes attached to each substantive theme ranged from four to eighteen subthemes. Many sub-themes overlapped and held shared meanings as I began to make connexions between the emergent data (Culvin, 2019).

With respect to this study, it was upon the conclusion of data collection that the second author contributed to the analysis of the data, as the larger project was finalised, and the lead researcher began to dissect the data set into specific key elements. With the aims of this study in mind, the data collected in relation to working conditions and maternity was anonymously shared with the second author for an additional stage of analysis, where initial subthemes were pulled together into coherent themes related to maternity. This thematic analysis completed by both researchers identified three themes that explain the experiences of professionalisation on the lives of women footballers in relation to maternity. These themes are as follows:

1. Employment contracts and maternity policy
2. Navigating professional football and motherhood
3. Motherhood and athletes' bodies

## Results

### Employment Contracts and Maternity Policy

All industrialised countries have work-family policies in place to support work-family reconciliation; however, the nature and extent of support vary greatly between countries and organisations (Hegewisch and Gornick, 2011). The United Kingdom introduced its first maternity leave legislation through the Employment Protection Act in 1975 (Jarvis, 2011). However, in relation to the contractual maternity policy in professional women's football in England, most players indicated that maternity is low on the list of priorities of clubs despite any legal and moral obligations. Data revealed the narrow understanding of players on the nature of their contract and the fine line between recognising how clubs protect themselves and their endorsers and how they advance the security of their employees. Players within this study were asked what type of contracts they were “on,” with 97% of players being unsure of the contract type and/or the support it does or should offer in terms of past economic remuneration. Indeed, within the data, one of the most problematic omissions in employment policy for the FA WSL footballers in this project centred on maternity and childcare support. Players discussed the neglect surrounding education, post-career planning, maternity, and childcare policies, including the following two players who were explicit in highlighting maternity pay and support as a necessary requirement:

*For me, I'd want private medical care, maternity leave - that should be standard. I would wait until after I'm done, cos I know I wouldn't get paid or looked after or get maternity pay. (Interview 5)*.

*No, I don't (have maternity in contract). My mum actually said the other day that like why aren't there any normal workplace polices for us as players? In the time I've been injured you could have had a baby and still got paid, cos I'm injured but not if I wasn't injured! You know, I'm injured so I'll get pregnant - what would they do then? (Interview 25)*.

This approach presents problems for players, as depicted in the following extract:

*But it's a contentious issue (maternity policy) cos like, if I'm coming to the end of my contract and then I'm like I wanna get pregnant like I wouldn't wanna lie to the club! Or are you honest and say I wanna have a baby and I wanna be part of the club? Like for the next year I'm out - once you've had that conversation does that change things? Would the manager just say, “well we'll sign you once your back fit and ready” Then are they allowed to do that? And you just sat there and said nothing which your entitled to do - then, I think people would be willing to sign the contract and then just drop the bomb in like 3 months' time! Cos at least they're guaranteed an income for the next two years! Cos you have a situation where you might not get a contract if your honest! There's no support in the meantime. (Interview 3)*.

For this player, the contradiction of short contracts and maternity poses a clear problem and demonstrates the challenges women footballers face in navigating their obligations to their clubs and their opportunity to have children. For other players, a lack of education on the issue of contracts and maternity policy was a concern, alongside the low prioritisation of maternity within clubs:

*Maternity is something I haven't been educated on and its only recently cos one of my mates has been pregnant… I don't know if we have it or not. We haven't had a meeting to clarify anything. (Interview 25)*.

*Education is key. I think we know now with maternity leave ends up in a big battle and the PFA had to step in with a player. She got some pay but can't imagine it was much. Oh yeah, I'd want that in my contract (maternity). The club just wouldn't be okay with that though, cos its money. The club see it as a waste of money. Cos with that money (maternity pay) they could bring in a player who can actually play. (Interview 8)*.

The Professional Footballers Association (PFA), established in 1907, is the world's longest established professional sportspersons union. The PFA states its experience ensures the best advice, representation, and assistance in all aspects of the career of a player and beyond. Whilst the PFA focuses on “sportspersons” and aims for inclusivity, a more nuanced understanding is generated through data of a partially visible union for women. On the question of PFA's support, it is clear that women footballers require employment policies specific to their own games inbuilt within contracts. Many of the players noted that their experience of employment is one bolted on to the men's game, with limited consideration for their employment needs. This is articulated by one experienced FA WSL player, who is also a player representative for the PFA, in her discussion of “selling” the benefits of the PFA to her fellow professionals:

*If you're a men's club, the PFA do workshops about post-career and life worries, I don't understand how they're still ‘working on it' (for FA WSL) we've had a professional league since 2011. Why is it taking so long? If anything, women need information about post-career, education, maternity. We need specific stuff, like if we get injured and your club can't support you, we need the PFA, yet they can't do anything for you! […] Although the PFA have done lots for the men's game they either don't offer enough to women or they don't make us aware (Interview 2)*.

In part, the lack of gender-specific contractual policies that players discussed can be explained by the professionalisation process being subjugated by a patriarchal composition (Woodhouse et al., 2019). The implication here is that processes of professionalisation have been overseen by, and mirrored on, men's football, whereby the cultural positioning of women and their employment needs are secondary. This was evidenced here:

“*The game is moving too fast […] we have too much reliance on the men's team that aren't doing well […] women become the scapegoat regardless of how successful we are” (Interview 13)*.

*You're so reliant on them doing well and staying up (men's team). Someone coming in charge who actually wants to invest in a women's team. There's so many if buts and maybes that that is a downfall. There's so much uncertainty. (Interview 3)*.

Despite this, for professional women footballers, contractual agreements are now part of the participatory process, yet, it is clear that omissions and inadequacies in employment policies exist in the contracts of these professional women footballers.

The relationship between male-dominated workplaces, such as football clubs, and women can be considered complicated. Women who enter a career as professional footballers face considerable challenges not least on the basis of legitimacy. Football development in England indicates an intimate relationship between the performance of gender and the cultural legitimacy of a restrictive female or male dichotomy (Themen, 2016). It could be inferred that this cohort of professional women footballers has embodied the gender order and as a form of symbolic power that influences behaviour. It appears taken-for-granted that players are uninformed and are seemingly reluctant to question their conditions regarding their employment rights, in relation to maternity policy. This may lead, perhaps unsurprisingly, to increased uncertainties at work. Thus, football, as a male-dominated sport, increases complexities for women as they bid to forge a career as a professional footballer. It is perhaps overly simplistic to assume the implementation of maternity policies would lead to women footballers utilising policy as exemplified in the following extracts:

“*But at this moment in time, it's not particularly great (at club). Obviously, things could be better but it's just I've been young, and you know the WSL is pro now… I've been a bit naive really. Cos you accept things cos you think it's a bonus when really, it kinda like… your like, ‘oh hang on a minute!' In a normal job that wouldn't happen” (Interview 27)*.

“*One of the girls (teammates) is having issues at our club, said she didn't wanna kick up a fuss in case they take her off her contract” (Interview 30)*.

Extracts of this type were not uncommon. Players have a heightened awareness of their improved situation through professionalisation and appeared, at times, discouraged and disempowered to voice contractual concerns because of their fear of rocking the boat and compromising their professional status. It seems evident that symbolic forms of masculine domination were naturalised and normalised to the extent that they are unrecognisable and are resisted to a point of possibility in sociocultural constraints (Dillabough, 2004). In this way, players highlighted gender-specific contractual and employment decisions related to their careers, namely, maternity and motherhood. This is not unusual, with Taylor et al. (2020) and Pavalidis (2020) reporting that paid sportswomen are grateful for the opportunity to play, which can lead to experiences of exploitation or concerns for speaking out against issues.

### Navigating Professional Football and Motherhood

Pregnancy and motherhood can impact the career of an athlete in complex ways (Appleby and Fisher, 2009), and, in sum, many participants felt that being an athlete-mother was incompatible with being a professional footballer. It was not uncommon for players to be unsure of their rights as potential and current mothers or, in the majority of cases, state the absence of maternity and/or childcare support from clubs, specifically in relation to their professional contracts. This contributed to the perception that maternity was problematic for both players and clubs, exemplified in the following extract of an experienced international:

“*How long clubs pay you for is another matter (if maternity was a contract policy)! I don't think there's enough information out there, for players getting pregnant and maybe that's not the clubs' fault cause, they don't necessarily know themselves. Cause it's not that common. It's something clubs need to get a hold of, cause, men don't need time off, but we do!” (Interview 24)*.

This extract exemplifies how this player has internalised a neoliberal shift from organisational to individual responsibility (Davies, 2005). By shifting the blame of the clubs onto the player, this player normalises her experience and disempowered position as common sense. In their FA WSL analysis, Woodhouse et al. (2019) suggest that such individualisation is a common strategy for minimising the gendered structures of an organisation. The combination of short-term contracts and the precarious nature of work makes pregnancy or maternity a highly problematic decision for elite women footballers, as exemplified in the following extract:

“*Never even thought about that, that's not in anywhere (maternity). I didn't even think about that but like, imagine if I got pregnant! Like you have to put something on hold that's natural thing cos the contracts are so short or … like, the England contract is yearly, my club contract is up soon as well. If they want me then I'll renegotiate if not, nothing, I'd leave. England is the same if they want you then you're in if not … like well I haven't been selected for a year so in my eyes I'm … gonna be like out, so then what will I do? I couldn't live. And like you'd get told a month before that your income is halved?” (Interview 21)*.

Despite the legal obligation for policy to support women in their maternity rights, players recognise that the short-term contracts in which they operate do not support their needs in this way. Data suggest that players do not trust clubs to support them contractually in family endeavours, which appears to lead to a mistrust of clubs and staff as articulated by one senior international:

“*If I'm thinking of contract length and my future I wanna have kids at some point in my career and still play football. I need the security of maternity leave. […] You can be a woman, have kids and play football. But I'm not sure that exists. Like if he (manager)gave me three years (on a contract) and I turned around and said I was pregnant what would he (manager) do? We need longer contracts, so we feel secure! I shouldn't have to think I need to sign a four-year cause, I wanna have a baby, so I know they'll pay me. I wanna be honest to my coach […] I wanna have kids and carry on playing (Interview 15)*.

It was not uncommon for players in this study to describe similar circumstances. It appears taken-for-granted that women footballers will not have children whilst they are playing competitively, as exemplified by a senior international:

*Literally, no idea (whether maternity policy is included in the contract). I mean, I think it's cos of the environment they don't expect things to happen like that? They do happen. Same as pregnancy - like we're women, some of us are gonna get pregnant, that's how it works. And I just don't think… especially, and I don't wanna disrespect, but in a male environment, they don't expect that. They don't think of the worst. Then when it happens it's like ‘shit I didn't expect that!' and it's like, well half your team have boyfriends that have been together for years, some way down the line that's gonna happen and that's how life is. (Interview 17)*.

These notions concerning the cultural expectations of motherhood appear to have been internalised by players and accepted as common sense. A discourse that circulates certain meanings attached to motherhood becomes difficult to challenge as it is inherently tied to gendered ideologies, such as that the true calling of a woman is to have children (McGannon et al., 2018). However, there appears to be an incompatibility between motherhood and professional football as an occupation, due to structural constraints around inadequate and precarious contracts and cultural constraints of the expectations of players. It was not surprising then that motherhood was not something all players considered.

Still, despite the mixed feelings towards the compatibility of their career and motherhood, it is clear that the omission of maternity and childcare policy within club contracts is highly concerning:

“*I mean yeah, like, if players wanna have kids it would massively help (having maternity policy). Obviously, you don't want people taking the piss, but I think it would help people's mentality if they knew they were supported in having a family. The word support lacks a little, it's a bit cutthroat in our game. And I know you have to be serious, and you can't treat players differently but there has to be a degree of flexibility in some cases. And you would like to think the human side of people, there would be flexibility and understanding but it's not the case sometimes. The problem is sometimes it's on the surface as well, they say they understand if you're a little late or something … then you're dropped! I feel like being a mum really held me back at XXX club and I wasn't offered the same contract as other players, purely cos I was a mum. I'm sure of it. They never said it obviously, but I would say 95% sure that if I didn't have a baby, then I would have got a better contract.” (Interview 24)*.

It is worth noting here, since 2018, central contracts issued by the FA to 30 elite senior internationals have maternity cover as a policy and contracts are renewed annually. Therefore, on the one hand, central contracts offer maternity cover and some security, whilst on the other, a key problem is the restrictive capacity of annual renewal. Annual renewals based on performance could actively discourage players from considering children due to the short-term nature of the contracts and their emphasis on performance. This is demonstrated in the following extract by a senior international:

*They're favourable towards the FA (contracts), they're careful to make sure you are not employees cos that means you have employer rights. We're paid Per Annum we now have maternity and holiday (central contract). Whereas with club you're an employee. What they don't want at England, they don't want you to have the rights. Puts you off getting pregnant. I need to check maternity at club explicitly. Cos we're at a club like XXX, we would. (Interview 10)*.

In sum, it is clear that professional women footballers are operating in an occupation that offers them very little security and is tangled up in contracts whereby they are considered as assets. There is a triad of concerns here. First, a consideration of why women appear complicit in their own subordination. Second, understanding whose responsibility it is to offer moral and legal security to professional women footballers. Finally, examining the consequences of insufficient contractual policies to support players. A final point of consideration in relation to motherhood is perceptions of players on the physical impact of pregnancy on their bodies and the implication of that on their careers.

### Motherhood and Athletes' Bodies

Whilst professional football contracts and policies were described as incompatible with motherhood, for some, the perceived mismatch of motherhood and athletehood were related to the impact on their bodies. Moreover, the gendered practical considerations of an occupation imbued by long periods away from home, inflexible working hours, and little control over relocation are all further evidence of a perceived incompatibility. This is exemplified by the next extract by one senior international:

“*I guess there's fear of not being able to get back where I am before I have kids, being fit. The fear of not getting a contract again. There are too many pressures you know? Babysitters, childcare and we're always away!” (Interview 13)*.

Some interviewees acknowledged concerns with losing their “feel for the game,” that is to say, perceived diminished capital through an extended period out of football. Within the field of professional football, research suggests that physical capital is of particular value (McGillivray and McIntosh, 2006). The techniques and highly sought-after physical competencies (Adkins, 2003) of elite professional footballers, for example, speed and strength, are accorded as the dominant capital. A handful of players considered motherhood similar to having a long-term injury or medical condition, and perhaps a reduction in the forms of embodied capital. The feelings of players towards motherhood ties in with previous research, which argues that athletic identity is so strong, athletes believe motherhood to be incompatible with their careers (Douglas and Carless, 2009).

“*The thing is can you get back? Will they give you another contract? It's something I haven't worried about yet, but it is a worry. But it isn't my priority, but maternity should be in there - cos we will never know what will happen!”(Interview 14)*.

The professionalisation of women's football in England has meant that demands placed on players have increased significantly in recent years (Datson et al., 2017). However, beyond the prestige and celebrity attached to playing professional football (Roderick, 2006) lies a darker dynamic of overexposure to elite sports, such as disordered eating, a problematic body image, and burnout (Hughes and Leavey, 2012). Football as a sub-field of sports has implicit blueprints that define and evaluate the ideal body types of skilled and committed participants. Haynes (2008) suggests that professionals attach meaning to their bodies as they symbolise identity, in this case, a professional athlete. This is demonstrated through the scrutiny players give to controlling their bodies and their lives at work and outside of work. The attention given to their bodies and how such focus can control players is detailed in the next extracts from two internationals:

“*If you get a comment on your weight or people say: ‘you don't look like you play football' people take offence yeah. Players feel as though you have to look athletic - pulling their shorts up to show massive quads or whatever. Players feel pressured to do it. Look at men, you can instantly tell they play, muscly with a six-pack […] women have to work harder, perhaps too hard cause they wanna prove a point, like they can look good” (Interview 22)*.

This example was typical of players who discussed their ambition to look athletic, “I think we have to try and look athletic […] myself I wouldn't wanna look heavy and non-athletic” (Interview 28). It was clear that self-regulation occurred as a response to looking athletic. “Looking the part” demonstrates the importance players attribute to legitimacy in the football field and how they embody their new career, giving further credence to the perceived incompatibility of motherhood as women typically report average weight retention of 0.5 to 4 kg, a year after pregnancy (Bø et al., 2016), although elite athletes are more likely to regain their pre-pregnancy body weight (Bø and Backe-Hansen, 2007). Embodied careers include feedback from the body around appropriate levels of physical and symbolic capital, which will enable both legitimacy and capital exchange, which is in stark contrast to pregnancy and returning to full fitness.

The central point is that players are expected to deal with high levels of intense scrutiny and loss of control over their bodies. It became evident in the data that players accept this intense approach to accountability. One predominant area of concern with the highly regulated culture of surveillance is the control over athletes' bodies and personal freedoms. The following example from a senior international highlights control over players' bodies and the potential consequences for individuals:

“*Physically we are made to feel like we have to be a certain way, a certain body shape to be accepted […] it's worrying when your sat in a dining area and the whole team won't have carbohydrates after an intense session cause, carbs are the ‘devil'. I dunno where it comes from but when your forever being fat tested, over tested. Clubs are aware and they're the ones who want it. At (club) we're told we have to be a certain body fat or you're in fat-club and you have to train by yourself!” (Interview 13)*.

The imposition of body fat testing, body shaming, the treatment of players' professional bodies, and its effects are exemplified in the next extract from a youth international:

“*We do body fat testing and the information got sent to the board! How is that information getting sent to the board? They wanna know that the investment is worth it! […] I'd put on a little bit but was playing better but the coach had to write an email to the board saying why I had put on x amount of fat” (Interview 4)*.

Many of the interviewees noted that body image was an issue for them and/or some of their teammates. Within the sporting environment pressures to be thin permeate and can encourage an unhealthy preoccupation with weight in athletes (Papathomas et al., 2015). Women athletes, in particular, are more susceptible to problems with body image (Roberts, 2018). Women participating in sports not only have to contend with broad cultural pressures to be slim, but additional pressures to conform to a particular body type that relates to their sport (Roberts, 2018). In the case of women athletes, the taken-for-granted is the incompatibility of motherhood and athletehood. Thus, it appeared that professional women footballers disciplined themselves in accordance with modes of normative femininity. This form of self-control becomes particularly problematic when we consider the biological and physical shift women experience throughout their pregnancy and post-partum. Perception is important to body image, when an individual perceives their body not to correspond to ideals, for example, athletic or feminine ideals, they have a greater probability of manifesting body image concerns (Sobrino-Bazaga and Rabito-Alcón, 2018). Evidence suggests that athletes who experience body image issues throughout their career are likely to continue post-career terminations (Papathomas et al., 2015).

## Discussion

This study has sought to provide evidence for the distinctiveness of career development and the gender-specific contractual conditions and requirements of women entering the profession of football. This involved identifying the contractual necessities of professional women footballers in England specific to maternity. In summary, women's football in England has undergone significant transformations in the last decade. Arguably, the most significant development is the reorganisation of the game from amateur to professional, creating the opportunity for football as work for women. This study has presented the voices of professional women footballers and their distinctive experiences at work in relation to contracts, maternity policy, motherhood, and their bodies. Specifically, data highlighted the incompatibility of motherhood in professional women's football in England. Women footballers are not afforded the same luxuries as their male counterparts in terms of contractual security, employment conditions, and economic renumeration. In this way, ambiguity exists between the growing glamourisation of women's football and the precarious work conditions in which players operate.

As previously discussed, women footballers have reported a lack of support with regard to maternity and childcare (FIFPro World Players' Union, 2017; McElwee, 2020). The problems for women footballers concerning maternity and childcare support appear 2-fold. In the first instance, women are operating in a sport that is almost exclusively dominated by men in its administration and organisation of sport (Williams, 2006). The second and related problem, evidenced here, appears to be the perceived acceptance of players and complicity of their position. From one situation to the next, one player to another there appeared to be an *ad-hoc* and arbitrary approach to contractual agreements of women.

We found that maternity and parental policy have been significant omissions in the contracts of professional athletes in the FA WSL in recent years unless an athlete was one of the 30 England squad players, then contracts were performance-based, 1-year rolling contracts. Predominately, we found that the women interviewed were unaware of what contracts should offer beyond economic remuneration and players felt that clubs placed maternity and parental support low on the list of priorities. What emerged within the data is that players simultaneously accepting the significant policy omission whilst recognising maternity policy is essential to a group of women at work.

Data revealed that players specified that maternity policies were not prioritised within their clubs. We consider this discriminatory process as an outcome of male-dominated governing bodies and clubs, which allowed the overlooking of the significant biological differences of women and the failure to accommodate them within their employment conditions, such as contracts. The women interviewed recognised themselves to occupy lesser positions in the field; therefore, they subscribed to the legitimacy of the principles according to their position (Schinkel and Noordegraaf, 2011). To this end, we concluded that the women in this study have normalised dominant practises. Hence, it can be proposed that the secondary position of women in the professional football field have been embodied in early socialisation experiences, meaning football as work is often reinforced and accepted as the job of a man and, as such, the needs of women are neglected, yet women accept this cultural positioning.

Whilst this analysis offers an accurate representation of the experiences of women in professional football, up to 2018 at least, it is also crucial to point out that, if maternity and parental policy were part of player contracts, it is not certain that women would utilise the policy. Concerns over their bodies were very real, which further highlights how athletes view motherhood and athletehood as incompatible. This finding correlates with and supports previous research (Dixon et al., 2008; Burton, 2015) and, despite a growing trend in gender-equitable policy and the increased verbal and symbolic prioritisation of policies including those in relation to maternity in football (FIFPro World Players' Union, 2020), we concluded that intent and action were potentially incompatible. More concretely, in line with Kirby and Krone (2002), it can be argued that widespread organisational change is not achieved automatically or simply by implementing gender equity policies. We suggest that it is crucial that men's teams who have absorbed women's teams must adapt to and accommodate women to ensure success both on and off the field. It cannot be assumed that there is one solution to achieve policy targets, as a one-size-fits-all approach does not unpick the varied nature of existing barriers (Shaw and Penney, 2003).

It can be considered that this lack of workers' social and political rights at work, specific to maternity, is highly gendered and problematic in an already precarious and uncertain career for women. However, professional women footballers are not a homogenous group of despondent workers, rather, conditions varied from club to club and with international status. Our analysis of professional football as an occupation for women, contracts, and policies clearly highlights a vast improvement in conditions, as players can now be considered professional footballers, yet a field for women that is still emerging and adapting to their gender-specific needs.

We wanted to conclude this study by providing evidence to shape gender-specific policy and contribute to more gender-equitable approaches, especially in relation to maternity, in elite sports. Considerations about maternity often align with broader discussions of gender equity policies, which have been widely adopted as an organisational value in many institutions across the world. Despite narratives of supporting gender equity as an organisational value, there are fewer women in leadership positions, women also receive less remuneration and have lower operational budgets, and are promoted less (Dixon et al., 2008; Hoeber, 2008; Burton, 2015; Cohen, 2018). Despite the increased verbal and symbolic prioritisation of gender-equitable policies, Kirby and Krone (2002) argue that widespread organisational change is not achieved automatically simply by implementing gender equity policy. This idea is developed by Burton (2015), who suggests that a reason for the distinction between verbalisation and the application of gender equity policies is organisations creating policies for the wrong reason. To extend this point, Burton (2015) suggests organisations create gender equity policies to secure funding or as a politically correct way to create a positive public relation.

The dearth of attention paid to the issue of athlete mothers should not be considered indicative of its lack of importance within professional sports. In contrast, how we treat and consider pregnant, or soon-to-be, athletes through contractual policy and structural acceptance exposes an unresolved issue in sports more broadly the following: whether sports, still largely dominated by men, can accommodate the gender-specific needs of their women counterparts. Consequently, we suggest three specific ways in which women athletes can be included in professional sports. First, further research should be conducted more broadly by both researchers and organisations to explore and identify the best practises for professional women's teams. There are many examples of successful athlete mothers in football and beyond, so what are the conditions that have led to both their success and acceptance? Second, another way is the development of a gender-specific framework that includes expected contractual conditions, including the rejection of pregnant women athletes as individualised, one-off success storeys. In conjunction with this, providing education to elite sportswomen and those in positions of power on navigating motherhood in elite sports, including contractual obligations of both the player and club, is key. Third, the last way is the advocation of the compatibility of motherhood and athletehood through organisations, such as FIFPRO and individual unions and clubs.

Critically, this study highlights the current omissions and inadequacies of maternity policy in the contracts of professional sportswomen whilst also recognising that there have been, and will continue to be positive shifts. However, new strategies, such as the FIFPro World Players' Union's (2020) Player Pregnancy and Parental Management guide, do not deconstruct the real issues surrounding education, maternity, childcare policy, and post-career planning. This includes concerns over the lack of embodied capital on the football pitch and in the eyes of team managers and over fears women may have in utilising maternity policy. The evidence here provides data to support the need for gender-specific policies and a need to regulate that process in line with policy recommendations such as those provided by FIFPro. There are very real concerns for the diminished career prospects of professional women footballers who become mothers.

## Data Availability Statement

The raw data supporting the conclusions of this article will be made available by the authors, without undue reservation.

## Ethics Statement

The studies involving human participants were reviewed and approved by University of Central Lancashire ethics committee. The patients/participants provided their written informed consent to participate in this study.

## Author Contributions

This publication is part of Dr. AC doctoral thesis. Dr. AB supported further analysis of data and development of emergent themes.

## Funding

The authors were the recipients of internal funding from the University of Salford (£834 GBP).

## Conflict of Interest

The authors declare that the research was conducted in the absence of any commercial or financial relationships that could be construed as a potential conflict of interest.

## Publisher's Note

All claims expressed in this article are solely those of the authors and do not necessarily represent those of their affiliated organizations, or those of the publisher, the editors and the reviewers. Any product that may be evaluated in this article, or claim that may be made by its manufacturer, is not guaranteed or endorsed by the publisher.
